# Research on the impact of driving environment complexity on driving safety based on Driver Sky View Index (DSVI)

**DOI:** 10.1371/journal.pone.0347999

**Published:** 2026-05-08

**Authors:** Li Quan, Yuan Chen, Duoting Su, Ayin Yan, Ying He

**Affiliations:** 1 Faculty of Chongqing Jianzhu College, Chongqing, China; 2 Faculty of Architecture and Urban Planning, Chongqing University, Chongqing, China; Zhejiang Agriculture and Forestry University: Zhejiang A and F University, CHINA

## Abstract

With ongoing urbanization, driving environments have become increasingly complex, substantially increasing the volume of information drivers must process. To explore the impact of environmental complexity on driving safety, this study addresses the limitations of previous research in quantitatively analyzing multidimensional urban environmental factors. It introduces the Driver Sky View Index (DSVI), a visual field–based metric for quantifying environmental complexity. A method for extracting the sky view ratio was developed by integrating 3D street scene modeling with semantic segmentation, enabling a nonlinear mapping between DSVI and environmental complexity. A virtual reality simulation was employed to model nine scenarios across three road segments characterized by varying DSVI levels. Eye-tracking technology and a synchronized driving behavior recording system were used to capture visual and operational responses from 24 participants. The entropy weight–rank sum ratio (EW-RSR) composite algorithm was then applied to quantify the impact of DSVI thresholds on driving safety. The results indicate that DSVI significantly affects both driver behavior and visual characteristics, effectively reflecting how environmental complexity influences driving performance. Moreover, DSVI demonstrates a strong linear correlation with driving load (R² = 0.641, p < 0.05). Notably, when the DSVI value ranges from 0.241 to 0.397, a marked improvement in driving safety is observed. As an innovative quantitative indicator, DSVI not only facilitates the evaluation and optimization of urban traffic environments but also provides a valuable reference for enhancing driving comfort and safety.

## 1. Introduction

In the road traffic system, which comprises pedestrians, vehicles, roads, and the surrounding environment, drivers must continuously perceive and respond to dynamic traffic conditions [1,2]. The substantial volume of visual information to be processed often leads to increased visual load [3]. This visual information is largely shaped by the complexity of the urban environment [2,4]; in more complex traffic settings, higher informational density imposes greater visual demands on drivers [5,6]. Excessive visual load can cause adverse effects such as stress, distraction, and reduced attentional capacity [7], ultimately compromising driving safety. Therefore, maintaining drivers’ visual load within an optimal range is essential for safe driving performance [8].

Building on this understanding, driving load is shaped by visual information originating from multiple environmental sources. Such complex and heterogeneous input requires drivers to sustain heightened alertness and adaptability [9]. Research has shown that various elements of the road traffic environment—including road geometry, roadside greenery, traffic signage, and the surrounding built environment—significantly affect drivers. These influences can be categorized into the following key aspects:

(1) Road Design: Features such as road geometry [10,11], roundabout size [12], and the number of lanes [13] substantially influence on drivers’ perceived safety. In particular, larger curve radii or an increased number of lanes can elevate cognitive load, thereby compromising driving safety.
(2) Roadscape Design: Roadside greenery is a crucial component of the driving environment and has been shown to influence driver behavior significantly [10,14]. Well-designed landscape elements can reduce distraction, alleviate fatigue, and enhance visibility and recognition of the road, thereby playing a vital role in improving traffic safety [15].
(3) Traffic Signage: The clarity and consistency of traffic signs and signals can mitigate drivers’ cognitive burden [16]. However, an excessive amount of signage can lead to information overload [17], which prolongs drivers’ reaction time and decision-making processes. The resulting increase in visual load consequently elevates driving risks.
(4) Built Environment: The height and density of adjacent buildings exert a strong influence on drivers’ visual perception and psychological state [14]. In addition, dynamic roadside activities—such as those in commercial or residential areas—and visual stimuli, including advertising billboards, can readily divert drivers’ attention and consequently increase visual load [14,18,19]. Furthermore, the placement of roadside facilities such as gas stations and bus stops may disrupt traffic flow and influence drivers’ decision-making [20]. These effects, in turn, elevate the potential for traffic risks.

Existing studies demonstrate that various components of the road environment significantly affect driving safety. In quantitative research, scholars have proposed several key indicators to assess the influence of the driving environment on safety, including the color richness index, skyline variation index, street width, and street aspect ratio [21–23].

Yang Yunxing et al. [24,25] integrated road landscape metrics with sky openness in urban traffic settings. They employed the sky view ratio to measure road enclosure and applied visual entropy to quantitatively evaluate driver safety and comfort. Dinesh Mohan et al. [24] demonstrated that greater road width and block size were associated with higher traffic accident mortality rates. Mohan further identified four critical visual-field factors influencing perceived environmental safety: the proportions of sky, sidewalk, roadway, and vegetation. Among these, the sky proportion exhibited a linear relationship with safety perception.

In urban cores, environmental changes are primarily reflected in the evolving built environment [26]. From the driver’s perspective, this built environment is both complex and dynamic. Traditional approaches that rely on single indicators—such as enclosure or sky openness—to evaluate environmental complexity tend to oversimplify the issue. Conversely, employing multiple indicators to fully capture this complexity introduces significant computational challenges. At present, no established metric exists that effectively quantifies the complexity of the road traffic environment from the driver’s visual perspective.

Indices such as the Sky View Factor (SVF), information density, and enclosure metrics have provided valuable insights into environmental form and visual conditions. However, because they are derived from static viewpoints and abstract ratios, these measures cannot adequately capture the dynamic visual experience of drivers.

By contrast, the DSVI is explicitly designed from the driver’s forward-looking perspective, integrating multiple spatial attributes into a unified perceptual index. This design enables the DSVI to capture real-time visual complexity and to establish a measurable link between environmental form, driver visual load, and safety outcomes. In this sense, the DSVI extends beyond traditional static indices, offering a driver-centered, perceptually grounded, and behaviorally validated tool for evaluating the complexity of urban road environments.

In summary, driving safety is intrinsically linked to the composition and complexity of the surrounding environment. However, current research has yet to establish effective indices and systematic methodologies to investigate the relationship between drivers’ visual perception and environmental complexity. To bridge this gap, the present study proposes the DSVI, a novel metric derived from street scene extraction and image segmentation techniques. The DSVI enables a quantitative assessment of the complexity of urban road traffic environments. Furthermore, an evaluation framework based on the entropy weight–weighted sum ranking score (EMM-WSRS) method is introduced to examine and quantify the impact of environmental complexity on driving safety. By integrating quantitative modeling with perceptual analysis, this study offers both theoretical insights and practical implications, providing a novel pathway to optimize urban road environments and improve traffic safety.

The remainder of this paper is organized as follows. Section 2 introduces the concept of the DSVI, its definition, and the process of quantifying road traffic environment complexity. Section 3 describes the experimental design, including the construction of simulation scenarios, participant recruitment, and data collection methods. Section 4 presents the results and discussion, focusing on driver reaction times, visual behavior, and the visual load evaluation model under varying DSVI conditions. Finally, Section 5 summarizes the main conclusions, highlights practical and policy implications for urban road planning and safety management, and outlines limitations and directions for future research.

## 2. DSVI for quantifying the complexity of road traffic environments

### 2.1. Definition and composition of DSVI

In this study, driving environment complexity is conceptually defined as the combined effect of geometric enclosure and element density within the driver’s forward field of vision. This baseline is shaped primarily by buildings, greenery, and roadway boundaries.

Existing evidence indicates that enclosure and element density constitute the most critical baseline factors, as they fundamentally determine the extent of visible space and the volume of information entering the visual channel. Empirical studies consistently show that narrow lanes, tunnels, and high roadside density are directly associated with elevated crash risk and behavioral adaptations such as speed reduction and lane positioning. By contrast, visual clutter (e.g., advertising signs) and unpredictability (e.g., vulnerable road users) function as amplifiers, imposing additional situational demands on top of this spatial baseline.

The DSVI is therefore positioned as a salient representation of the spatial–visual dimension of driving environment complexity. By quantifying the proportion of visible sky in the forward field of vision, it integrates geometric enclosure and element density into a single tractable measure. It does not claim to comprehensively capture all facets of complexity (e.g., dynamic traffic, weather, or social interactions), but rather focuses on the spatial–visual baseline that consistently governs drivers’ perceptual load and safety outcomes.

**Fig 1:**
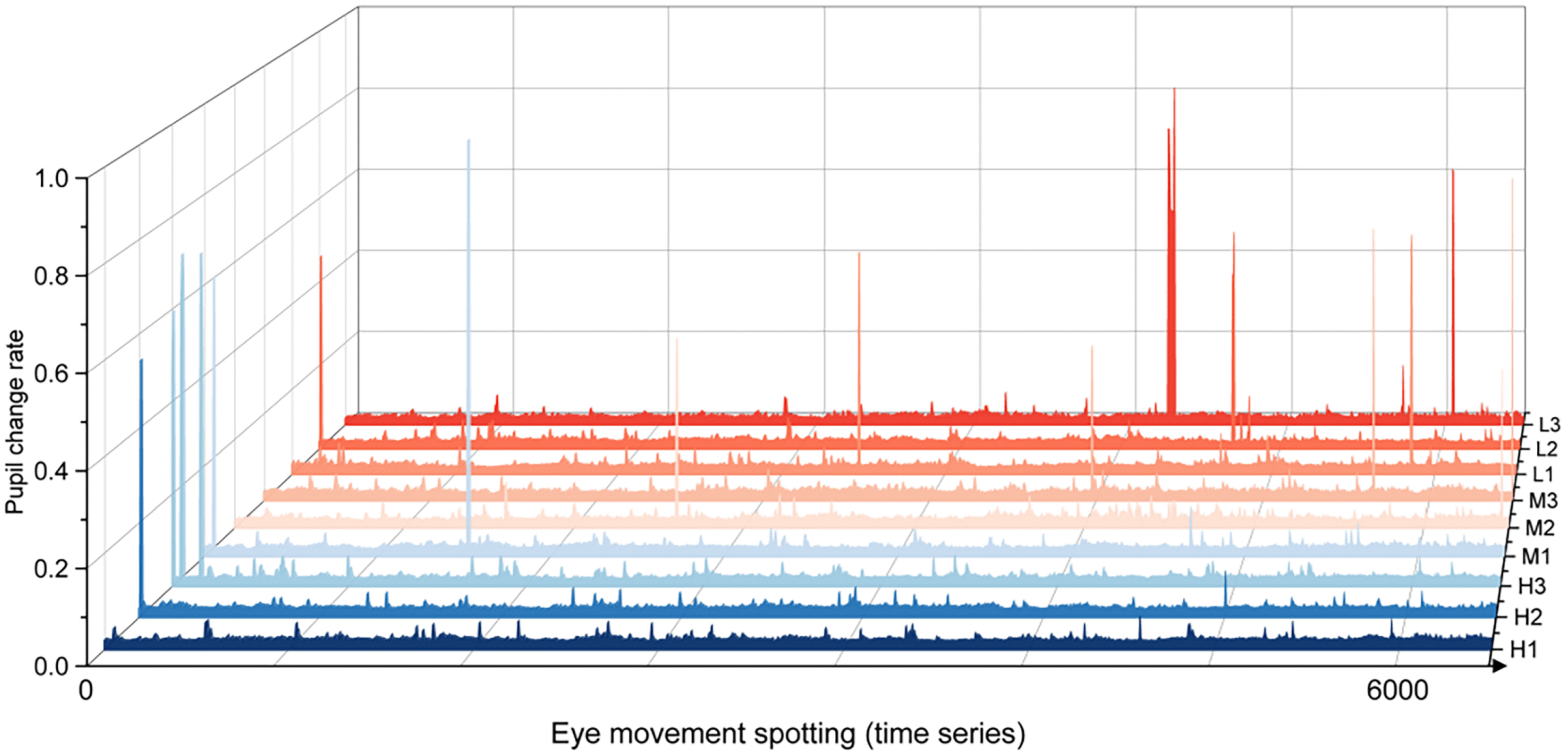
Components of the DSVI.

In the spatial dimension, DSVI captures the dynamic visual cone directly in front of the driver—that is, the continuously shifting field of view encountered during driving (Fig 1a). In the environmental dimension, it incorporates key spatial attributes, such as green coverage ratio, floor area ratio [27], building density [28], building height [29], building layout [30], and street aspect ratio [31]. Through semantic segmentation of driver-perspective images, the sky region is extracted (Fig 1b). The proportion of visible sky area is ultimately used as a surrogate for the visual load experienced while driving.

The mathematical formulation of DSVI is provided in Equation (1):


$$\text{DSVI}=\left(\frac{A_{sky}}{A_{total}}\right)\times \text{100}\%$$ (1)

Where Asky is the segmented sky pixel area, and $A_{\text{total}}$ is the total pixel area of the visual field.

### 2.2. Acquisition of DSVI

**Fig 2:**
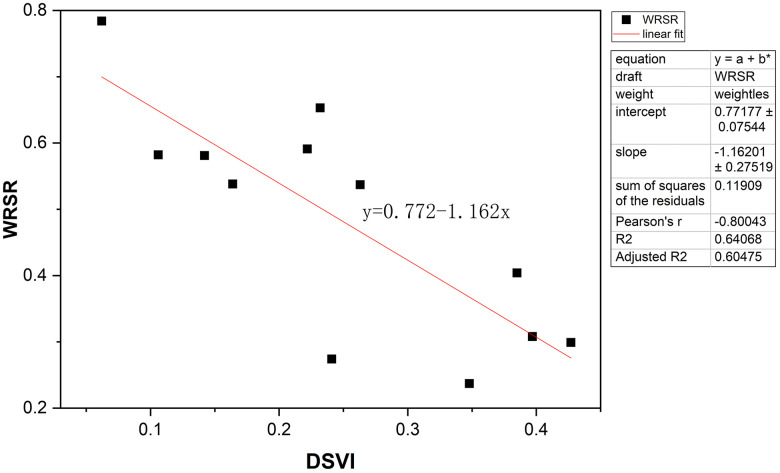
The process of obtaining DSVI.

The core contribution of DSVI is the quantification of the spatial composition of the road environment as perceived from the driver’s visual field. In this study, DSVI is derived through four key stages: road data acquisition, street-view image processing, environmental component extraction, and data validation. The overall workflow is illustrated in Fig 2.

(1) Street View Data Acquisition:

Based on vector data of the road network in the main urban area of Chongqing, major road segments were mapped using GIS technology [32], and street view sampling points were generated at 100-meter intervals. A Python script was employed to access the Baidu Street View Map API and retrieve forward-facing images with a 90° horizontal field of view, simulating the driver’s perspective. The images were acquired at a resolution of 1024 × 768 pixels. The virtual camera height was calibrated to 1.2 meters based on field measurements, corresponding to the eye level of a seated driver.

(2) Image Semantic Segmentation:

The collected street view images were processed using a Fully Convolutional Network (FCN), a deep learning-based architecture for semantic segmentation. Key environmental components—including buildings, vegetation, sky, and road surfaces—were identified and segmented to enable pixel-level classification.

(3) Environmental Component Indices and DSVI Calculation:

RGB values of each semantic category were extracted from the segmented images and classified at the pixel level. Based on the proportional areas of building coverage, green view index, and sky view index within each image, the corresponding DSVI value was calculated.

## 3. Experimental design and method

This study investigates the impact of DSVI-based road complexity on driving safety by integrating DSVI value computation, experimental scenario construction, small-target recognition tasks, and eye-tracking–based visual behavior monitoring.

The study protocol was reviewed and approved by the Ethics Committee of Chongqing University. The research complied with the ethical principles outlined in the Declaration of Helsinki (1964) and its subsequent amendments.

All participants were informed about the purpose and procedures of the study and voluntarily participated. Written informed consent was obtained from all participants prior to data collection. For brief, low-risk procedures (e.g., short perception or evaluation tasks), verbal consent was obtained in accordance with institutional guidelines.

All collected data were anonymized during processing to ensure the confidentiality and privacy of participants.

### 3.1. Experimental environment and system parameters

In accordance with the standards for primary urban roads, a bidirectional six-lane roadway was designed, comprising motor vehicle lanes, non-motorized vehicle lanes, green belts, and sidewalks. The design conforms to the specifications outlined in the Code for Design of Urban Road Engineering (CJJ37–2012). The total road length is 4.5 km, with a design speed of 60 km/h.

The Yuzhong Peninsula area in Chongqing was selected as the reference site for deriving real-world data to support the simulated experimental environment. Street view images of local roads were collected and semantically segmented to extract visual scene components and calculate corresponding DSVI values.

**Fig 3:**
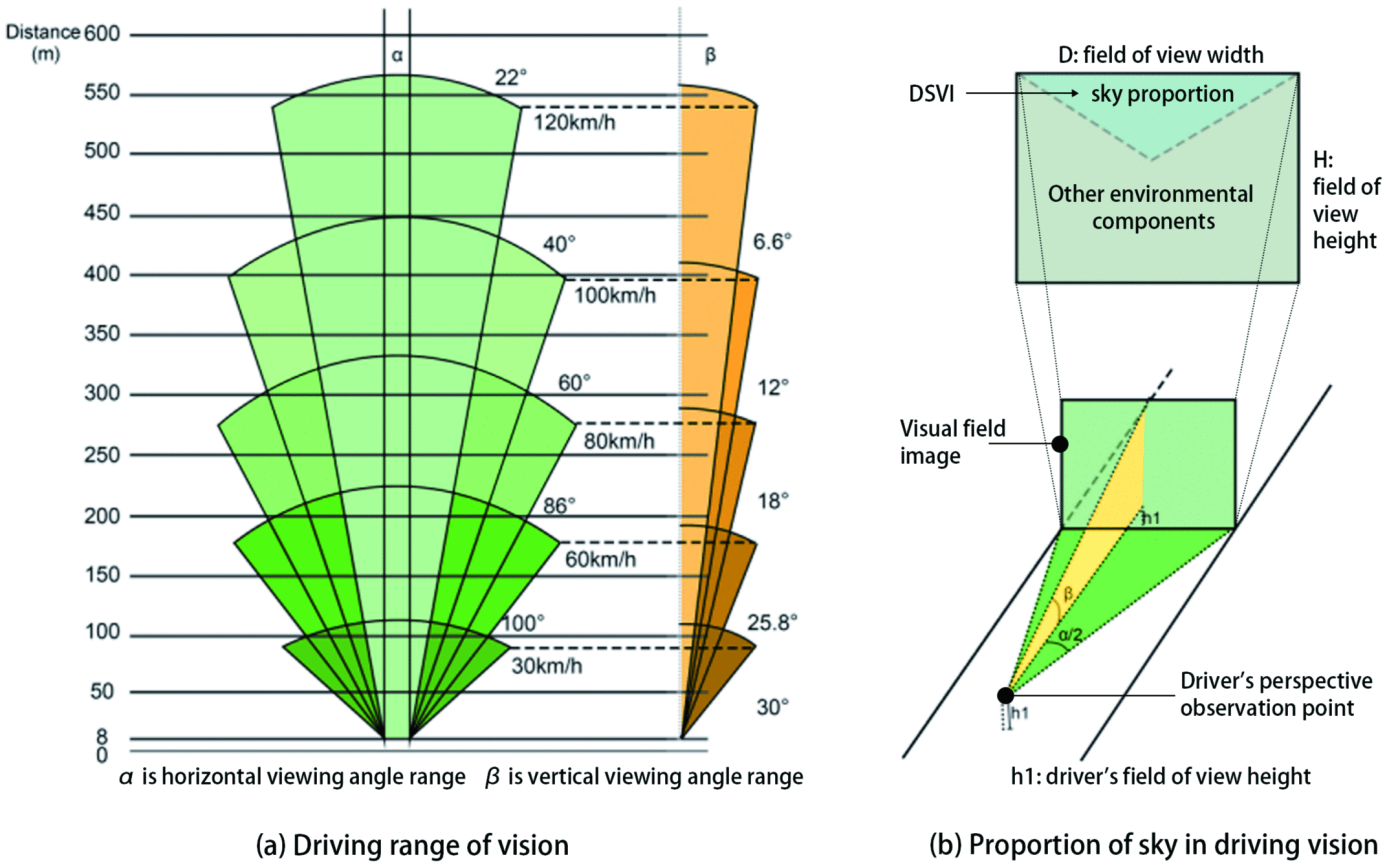
Driver’s perspective scene of experimental road segment nodes.

Based on the distribution of the surveyed DSVI values, three levels of road spatial complexity were defined to evaluate their effects on driving behavior. The DSVI was classified into three categories: high (H-DSVI), medium (M-DSVI), and low (L-DSVI). For each DSVI level, four observation points were selected across three representative road segments. Each segment was further divided into three equal-length sections. The segmentation approach and corresponding spatial nodes are illustrated in Fig 3, while the DSVI value ranges for each level are presented in Table 1.

**Table 1:** Range of sky field of view index values for drivers on experimental road sections.

H-DSVI	Value range	M-DSVI	Value range	L-DSVI	Value range
H1	0.427-0.397	M1	0.222-0.232	L1	0.164-0.142
H2	0.397-0.385	M2	0.232-0.241	L2	0.142-0.106
H3	0.385-0.348	M3	0.241-0.263	L3	0.106-0.062

### 3.2. Subjects and experimental tasks

To ensure the accuracy and consistency of experimental data, participants were rigorously screened for physiological conditions, behavioral habits, and adaptability to the experimental environment. Eligible participants were required to be in good physical health, well rested before the experiment, and have no history of major traffic accidents. To minimize external influences, they were instructed to avoid alcohol and caffeinated beverages within 24 hours, and refrain from strenuous physical activity within one hour prior to testing. Participants with myopia were required to wear lenses compatible with the eye-tracking device, while tinted or cosmetic contact lenses was strictly prohibited to prevent data interference. In addition, all participants passed a standard color vision test before enrollment.

After screening, 24 eligible participants (7 males, 17 females) were recruited. Their ages ranged from 20 to 32 years (M = 24.3, SD = 7.1). Driving experience ranged from 1 to 5 years, with a mean of 1.6 years (SD = 1.2).

**Fig 4:**
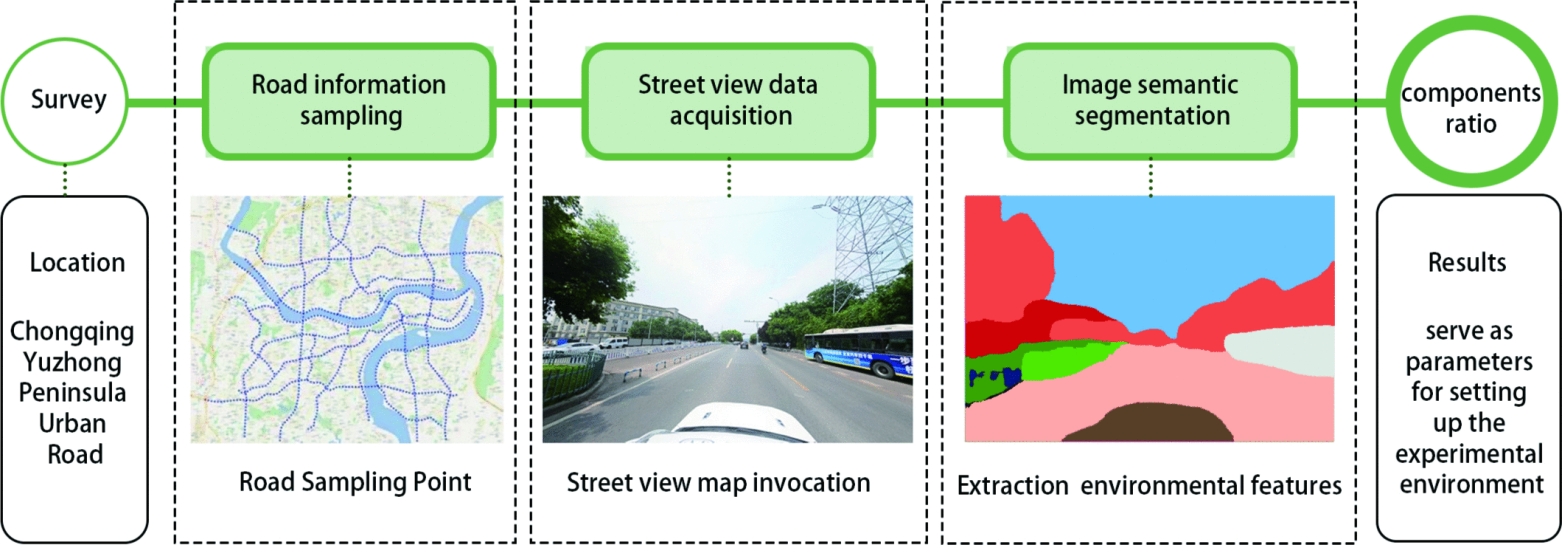
Experimental flowchart.

The experimental setup and procedure are illustrated in Fig 4. Before testing, all participants watched an instructional video and adjusted the display screen to approximate real-world lighting conditions. They then entered the laboratory in groups and underwent a 30-minute adaptation period to minimize the effects of sudden environmental changes. During the experiment, the laboratory was kept closed and access was restricted to avoid external light interference. Indoor lighting was provided by sources with a correlated color temperature of 6500 K, ensuring consistent ambient illumination.

A custom-developed reaction time testing system was used to record participants’ obstacle recognition and braking reaction times during the simulated driving tasks, providing synchronized outputs of obstacle onset times and spatial coordinates.Visual behavior was captured using a Tobii Glasses 2 wearable eye tracker, was employed, which enabled high-precision, real-time wireless data collection across scenarios. Experimental scenes were presented on a TCL 75Q10H monitor with a peak brightness of 3000 nits to provide sufficient luminance for realistic visual simulation.

### 3.3. Quantitative method for driver visual load

Statistical tests, including the Brown–Forsythe test, were applied to evaluate group differences. When significant effects were detected in the one-way analysis of variance (ANOVA), post hoc multiple comparisons were performed to further examine the impact of DSVI variations on driver reaction time.

In addition, pupil area was adopted as a physiological indicator to assess the influence of DSVI on changes in drivers’ visual responses [33,34]. The rate of change in pupil area was calculated as follows:


$$S=\frac{A_{t+\text{1}}-A_{t}}{A_{t}}$$ (2)

Where *S* is the rate of change of the driver’s pupil area; $A_{t}$ before is the size of the pupil area at the previous moment; $A_{t+\text{1}}$ is the size of the pupil area at the next moment.

To quantify the impact of DSVI on driving safety, an evaluation model was established using the entropy-weighted EMM-WRSR (Entropy Method–Weighted Rank Sum Ratio) approach.

Physiological parameters, particularly eye movement metrics, provide a direct and reliable means of assessing drivers’ visual load and are frequently analyzed with statistical methods to examine the relationship between visual responses and perceived workload.

In this study, four visual indicators and reaction time were selected as core evaluation variables. The entropy-weighted EMM-WRSR model was then applied to assess driving safety under different DSVI levels from the perspective of visual information processing.

The evaluation procedure consists of the following steps:

(1) Selection and Standardization of Evaluation Indicators.

$$X_{\text{min}}=\text{min}\left(X_{\text{1}j},\ldots ,X_{nj}\right)-\text{0}.\text{0001}$$ (3)

$$X_{\text{max}}=\text{max}\left(X_{\text{1}j},\ldots ,X_{nj}\right)+\text{0}.\text{0001}$$ (4)

$$Z_{ij}=\frac{X_{ij}-X_{\text{min}}}{X_{\text{max}}-X_{\text{min}}}$$ (5)

(2) Apply the entropy weight method to determine the weight of each evaluation indicator. First, standardize the data, and then calculate the entropy-based weight of the ith item under the jth visual evaluation indicator.

$$P_{ij}=\frac{\overset{\text{1}}{\underset{ij}{X}}}{\sum_{i=\text{1}}^{m}\;\;\overset{\text{1}}{\underset{ij}{x}}}(i=\text{1},\ldots ,m;j=\text{1},\ldots ,n)$$ (6)

Next, calculate the entropy value of the jth indicator e_j._


$$e_{j}=-k\sum_{i=\text{1}}^{n}\;p_{ij}\text{ln}\left(p_{ij}\right),j=\text{1},\ldots ,m$$ (7)

Where *k = 1/ln(n)*>0, satisfies *e*_*j*_ ≥ 0, the information entropy redundancy is *dj = 1-*$e_{j}$*,j =* 1,…,m*.*

The final weights are calculated as w_j._


$$W_{j}=\frac{d_{j}}{\sum_{j=\text{1}}^{m}\;\;d_{j}},j=\text{1},\ldots ,m$$ (8)

(3) Calculate the rank value. The formula is as follows.

$$R_{ij}=\text{1}+(n+\text{1})\frac{X_{ij}-\text{min}\left(X_{\text{1}j},\ldots ,X_{nj}\right)}{\text{max}\left(X_{\text{1}j},\ldots ,X_{nj}\right)-\text{min}\left(X_{\text{1}j},\ldots ,X_{nj}\right)}$$ (9)

$$WRSR_{i}=\frac{\text{1}}{n}\sum_{j=\text{1}}^{m}\;\;W_{J}R_{ij}$$ (10)

Let $R_{ij}$ denote the rank of the *j* th visual evaluation indicator at the ith observation point, and let $W_{j}$ represent the weight of the *j* th indicator, where the sum of weights satisfies $\sum W_{j}=\text{1}$. The Weighted Rank Sum Ratio $\left({\text{WRSR}}_{i}\right)$ reflects the overall performance of the ith evaluation object, with larger values indicating better performance.

Each visual evaluation indicator is ranked based on its magnitude, and the corresponding ranks $R_{ij}$ are used to replace the original indicator values. A rank data matrix is then constructed, and the WRSR value for each observation point is computed using the weighted sum of ranks.

(4) Tabulate the distribution of WRSR values and compute their corresponding Probit values. First, arrange the WRSR values in ascending order. For each group, calculate the cumulative frequency Σf, determine the rank *R*, and compute the cumulative probability as $p=\frac{R}{n}\times \text{100}\%$, where *n* is the total number of observations.

The final cumulative probability is corrected using $\left(\text{1}-\frac{\text{1}}{\text{4}n}\right)\times \text{100}\%$. Based on the cumulative probability values, the corresponding Probit values are then obtained from the standard normal distribution table.


Probiti=u(pi)+5 (11)

where u(pi) is the cumulative frequency, Probiti corresponds to the standard normal deviation.

(5) Establish a regression model using the WRSR distribution value as the dependent variable and the corresponding Probit value as the independent variable.
(6) Based on the fitted WRSR values, rank the visual load, classify it into discrete levels, and construct a regression model to analyze the relationship between DSVI and visual load levels.

## 4. Results

The experimental results demonstrated that variations in DSVI exerted significant effects drivers’ reaction times, gaze behaviors within areas of interest, and changes in pupil size across different visual environments. From the perspective of visual information processing, these findings provide a multidimensional evaluation of driving safety under varying DSVI conditions and offer new insights into how spatial visual complexity shapes driver performance.

### 4.1. Analysis of visual recognition of small targets

A total of 1,432 reaction time samples for small-target detection were collected. The distribution of reaction times approximates an exponentially modified Gaussian (EMG) curve, with frequencies gradually decreasing and stabilizing beyond 1,000 ms. Responses below 100 ms, typically reflecting anticipatory responses or rapid guessing, were excluded from analysis as they do not represent genuine reaction behavior [33]. Accordingly, this study focused on reaction times between 100 ms and 1,500 ms, with values exceeding 1,500 ms classified as indicative of potential driving risk.

Tests for homogeneity of variance were conducted using the Brown–Forsythe test. The results showed that the assumption of equal variances was satisfied in both grouping schemes (three-group division: p = 0.051; nine-node division: p = 0.447), thereby justifying subsequent one-way ANOVA for group-level comparisons.

#### 4.1.1. Differences in small target recognition.

One-way ANOVA revealed significant differences in small-target recognition times across road segments with varying DSVI levels (F(2, 1014) = 4.24, p = 0.015). Post hoc comparisons showed no significant difference between H-DSVI and M-DSVI segments. However, recognition times differed significantly between H-DSVI and L-DSVI (p = 0.006) and between M-DSVI and L-DSVI (p = 0.025).

**Fig 5:**
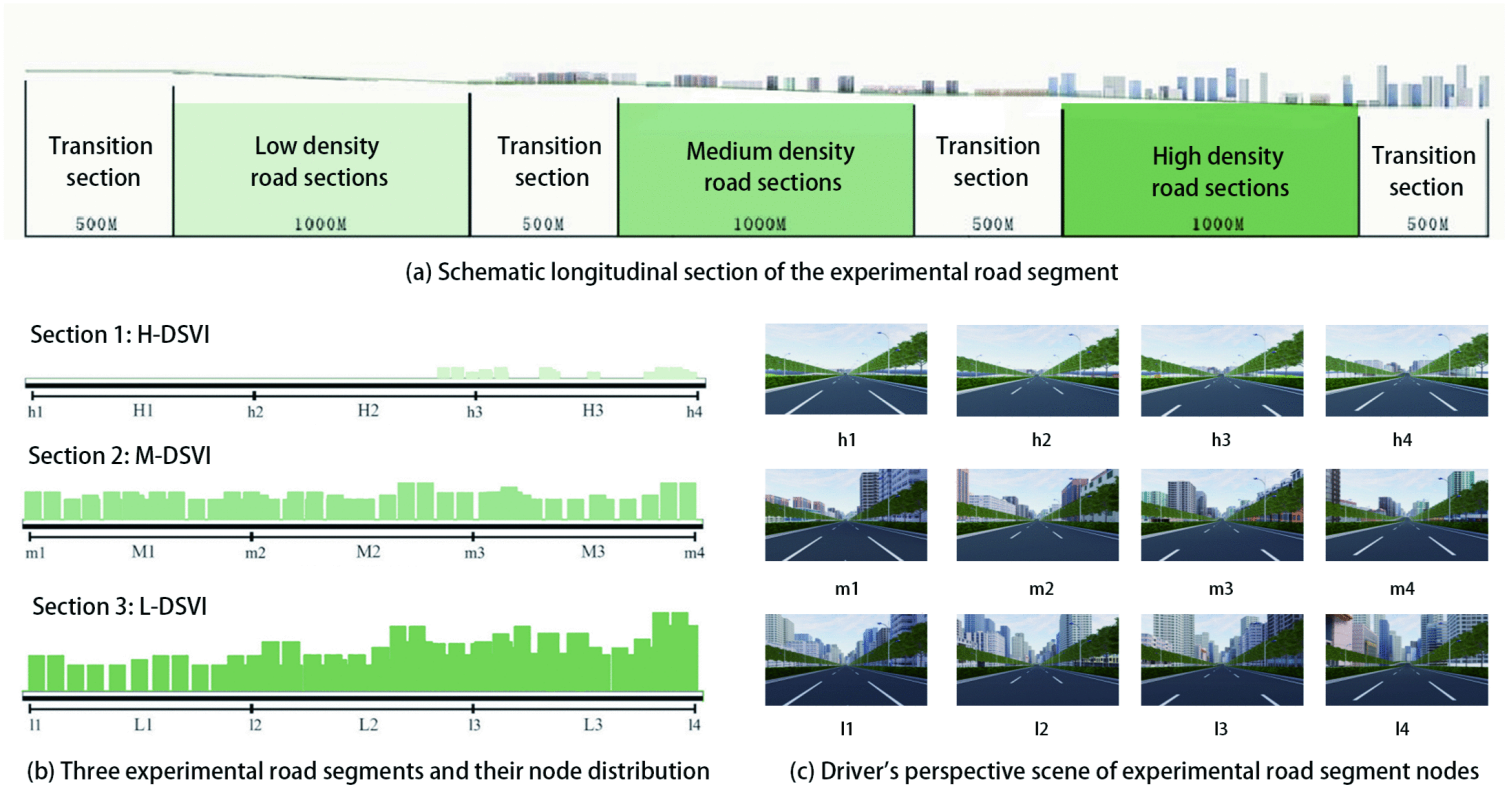
Box plot of reaction time in different DSVI sections.

As illustrated in Fig 5, drivers exhibited shorter and more consistent reaction times in M-DSVI environments, with reduced variance, indicating greater response uniformity. In contrast, the L-DSVI condition displayed greater variability and longer reaction times, reflecting reduced stability in target recognition. Overall, these results suggest that lower DSVI environments diminish drivers’ ability to detect visual targets consistently and efficiently.

To further clarify the influence of DSVI on drivers’ reaction times, a comparative analysis was conducted across different road segments. Variance analysis indicated that DSVI at the road interface had a significant effect on reaction times (F(8, 1014) = 2.718, p = 0.006 < 0.05), demonstrating meaningful differences across DSVI conditions.

**Fig 6:**
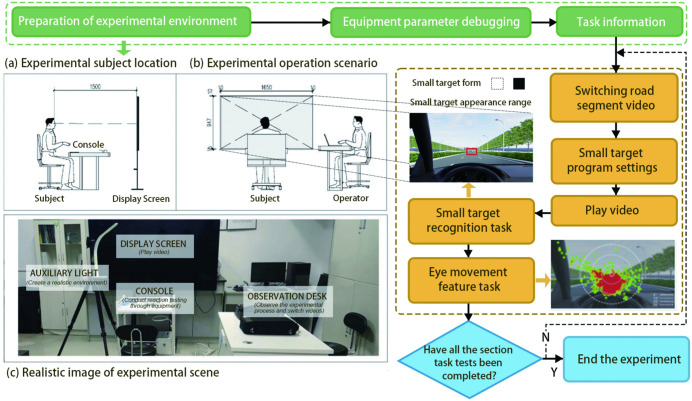
Box plot of reaction time at different DSVI segmented interfaces.

Further post hoc comparisons (Fig 6) revealed the specific effects of DSVI variations on driver performance. In the H-DSVI segment, reaction times decreased from H1 to H3, accompanied by reduced variability, indicating enhanced stability and efficiency in small-target recognition. A statistically significant difference was observed between H1 and H3 (p = 0.034 < 0.05), while the comparison between H1 and M3 showed a marginally significant difference (p = 0.069 < 0.1).

In the M-DSVI segment, as DSVI increases from M1 to M3, drivers’ reaction times progressively shortened, reflecting improved small-target recognition. No significant differences in reaction times were observed within the M-DSVI group. However, between-group comparisons revealed several significant differences, including H2 vs. L3 (p = 0.003), H3 vs. L1 (p = 0.023), H3 vs. L3 (p < 0.01), M1 vs. L3 (p = 0.011), M2 vs. L3 (p = 0.003), M3 vs. L1 (p = 0.050), and M3 vs. L3 (p < 0.01).

In the L-DSVI segment, drivers exhibited longer reaction times than in the other segments. Substantial variability was observed across the L1 to L3 sections, indicating inconsistent performance in small-target recognition with no clear within-group trend. At the lowest DSVI level, both mean reaction time and variance peaked, reflecting heightened difficulty and reduced stability in visual target detection under low DSVI conditions.

**Fig 7:**
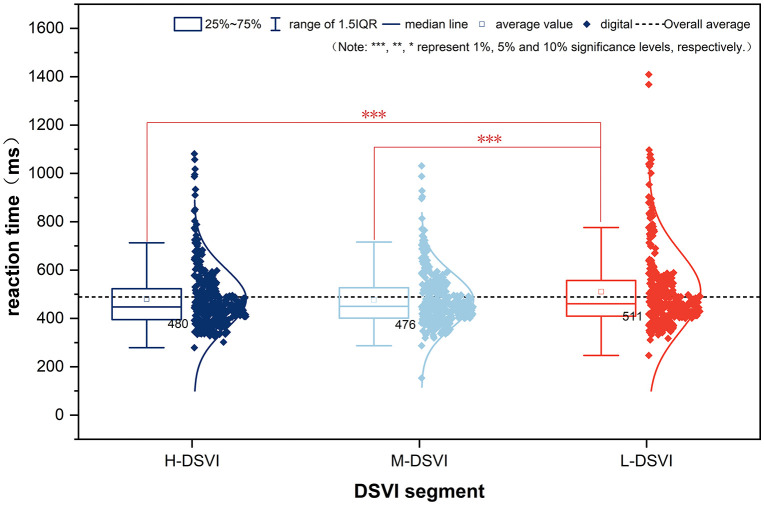
Plot of response time of drivers.

**Fig 8:**
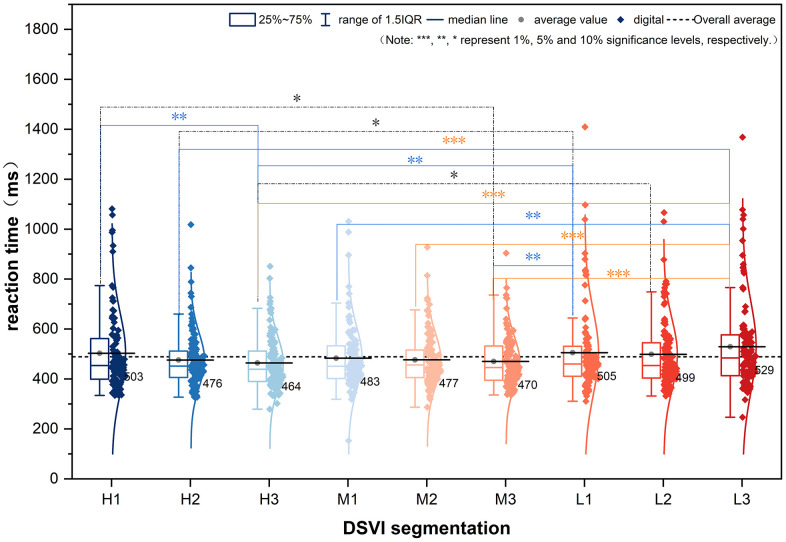
Schematic diagram of the danger point in each segment.

Reaction times for 24 drivers, arranged according to the sequence of small-target appearances, are presented in Fig 7. The results showed that reaction times were generally longer in both the H-DSVI and L-DSVI segments. Specifically, when a target appeared for the first time or when DSVI decreases sharply, drivers exhibited increased performance fluctuation, leading to reduced target recognition efficiency. As illustrated in Fig 8, average reaction times begin to decline upon entering the H2 segment, whereas in the M3 segment they remained above the overall mean.

A sharp spike in reaction time was observed at the beginning of each segment, followed by a gradual decline. This pattern suggests that sudden changes in DSVI initially impaired vdrivers’ visual processing efficiency, but reaction times gradually normalized as they adapted to the new visual environment.

The shortest reaction times were observed in segments H3 (DSVI: 0.385–0.348) and M3 (DSVI: 0.241–0.263), followed by H2 (DSVI: 0.397–0.385) and M2 (DSVI: 0.312–0.320). In segment M1, the DSVI ranged from 0.222 to 0.232. Collectively, these results suggest that optimal recognition of small visual targets occurred when DSVI values were within the range of 0.222–0.397.

#### 4.1.2. Dangerous point.

In this study, reaction times exceeding 1,500 milliseconds were defined as dangerous points, and their spatial distribution across road segments was analyzed (Fig 8). A total of 37 dangerous points were identified, most of which were concentrated near the road edges, particularly along lane dividers. White small targets accounted for thelargest share (n = 25). Across segment types, 17 dangerous points were recorded in the H-DSVI segment, 4 in M-DSVI, and 16 in L-DSVI.

Analysis of effective reaction time trends showed that upon entering the L-DSVI segment, drivers’ average reaction times increased, accompanied by a corresponding risein the frequency of dangerous points. Comparative video analysis suggested that the predominance of white small target dangerous points may have resulted from poor visual contrast with background lane markings. The H-DSVI, particularly H1, exhibited the highest number of dangerous points. This outcome may have been influenced by the open visual field and reduced cognitive vigilance during the early driving stages, which increased the susceptibility to visual distraction. In addition, unfamiliarity with the simulated environment at the beginning of the experiment may have further contributed to this effect.

In the M-DSVI segment, the visual presence of roadside buildings was observed to enhance driver alertness. As drivers had already adapted to the simulation environment during earlier segments, dangerous points in this stage were relatively few and mainly associated with subtle environmental variations or incidental distractions. This pattern corresponded with reduced average reaction time, although a small number of dangerous points persisted.

Upon entering the L-DSVI segment, visual complexity increased substantially. Drivers experienced heightened cognitive load and distraction due to the reduced sky view and more enclosed surroundings, which made target recognition more difficult. Although average reaction times rose in this segment, the number of dangerous points was lower than in the H-DSVI segment. This result suggests that despite the increased environmental difficulty, drivers demonstrated improved adaptability and task engagement, which helped mitigate the occurrence of dangerous points.

### 4.2. Analysis of drivers’ visual characteristics

#### 4.2.1. Field of view.

**Fig 9:**
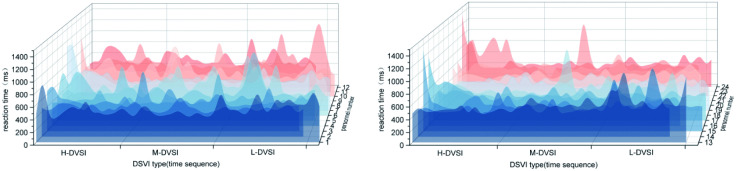
Visual field heatmaps of each node.

A statistical analysis of drivers’ visual behavior was conducted across 12 roadway nodes (Fig 9). Overall, drivers’ gaze was primarily concentrated within the central visual field, with the most fixation points located in the central and lower portions of the roadway. Between-group differences were evident. In the H-DSVI segment, the upper visual field (sky region) received relatively little attention, whereas under L-DSVI conditions drivers devoted greater attention to the upper field, including the sky, as visual information load increased. This shift indicates that drivers entering the L-DSVI segment adopted more active scanning strategies to obtain relevant environmental cues. Accordingly, fixation points partially shifted shifted upward the sky and adjacent buildings, and this trend became more pronounced with decreasing DSVI values.

From node M1 to L4, fixation heatmapsshowed progressively greater dispersion of gaze points, peaking at node L4. Combined with the small-target detection results, this pattern indicated that drivers in these segments allocated more attention to surrounding buildings. Such shifts in visual focus contributed to increased gaze dispersion, a higher incidence of missed detections, and longer average reaction times.

**Fig 10:**
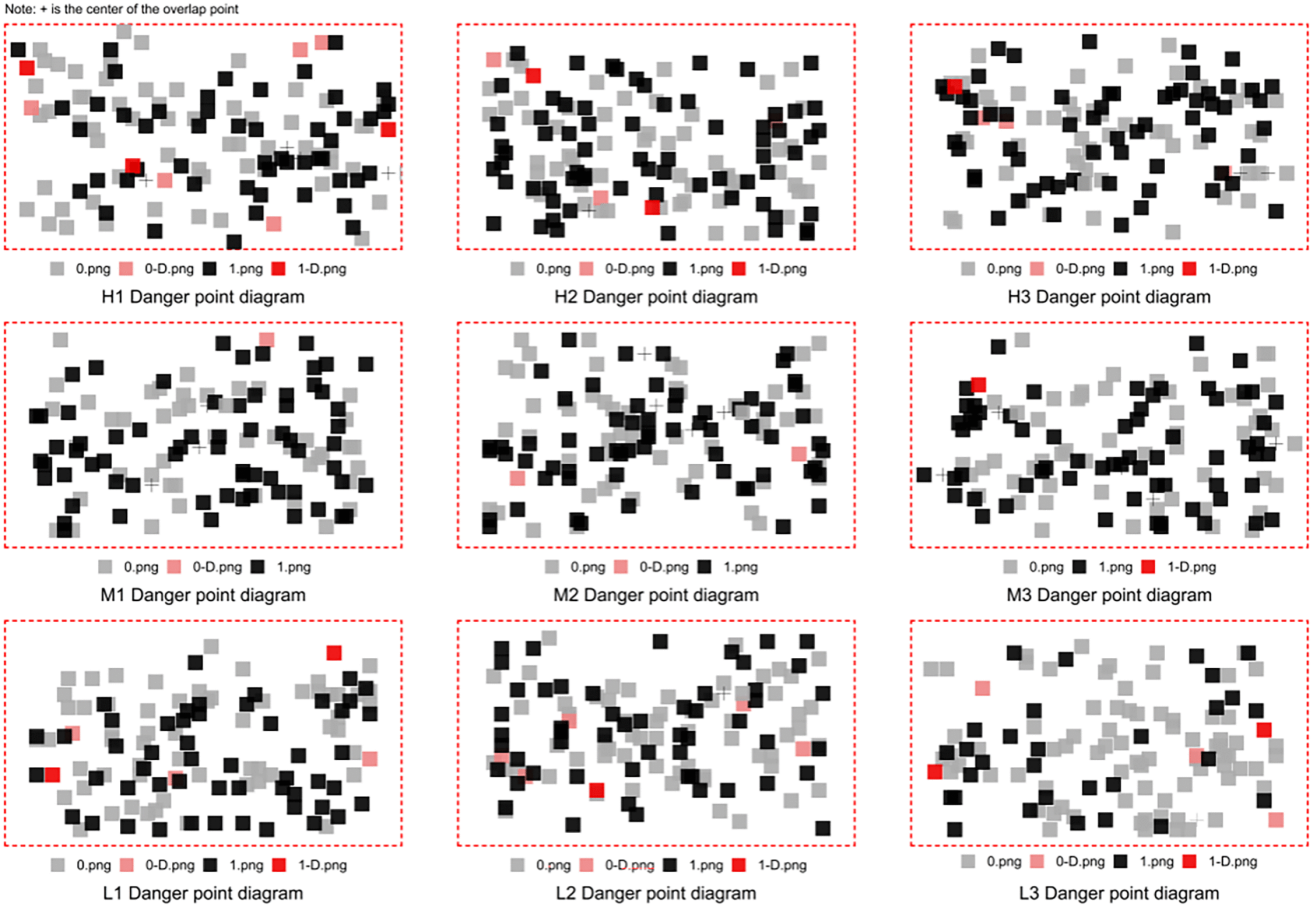
Analysis of drivers’ visual field gaze behavior.

During driving, drivers continuously perceive and process visual cues from the environment. Eye-tracking indicators such as first fixation time, fixation count, and fixation duration were used to assess the cognitive effort required to extract environmental information. As shown in Fig 10(a) and 10(b), fixation counts and durations were highest within the 10° and 20° fields of view (FoV), where drivers primarily focused on the road surface. Both metrics, however, exhibited substantial variability. Within the 20° FoV, fixation duration decreased as DSVI declined, whereas within the 10° FoV, fixation durations peaked at the end of H4 and M1. These findings suggest that drivers adjusted their visual strategies in response to abrupt changes in DSVI.

In both the 2° (narrow) and 30° (broad) FoVs, fixation durations and counts were markedly lower, with the 30° FoV exhibiting the lowest values across all metrics, which indicated reduced visual attention in peripheral zones.

Regarding first fixation time, the 20° and 30° FoVs showed minimal differences, suggesting rapid scanning behavior across broader visual areas. As shown in Fig 10(c) and 10(d), within the 2° FoV, targets were detected with longer latency but were followed by prolonged fixation durations. In contrast, within the 10° FoV, targets were detected earlier and fixated upon for longer periods throughout the driving task. Within the 20° and 30° FoVs, first fixation also occurred earlier; however, fixation durations were generally shorter.

From M4 to L4, fixation durations in the 30° FoV increased significantly, particularly between L3 and L4. This trend aligns with drivers’ adaptive behavior of monitoring central road conditions before reallocating gaze to the periphery. In more complex visual environments, drivers spent increased time observing the surroundings, which corresponded to a relative reduction in central gaze allocation.

#### 4.2.2. Pupillary responses.

**Fig 11:**
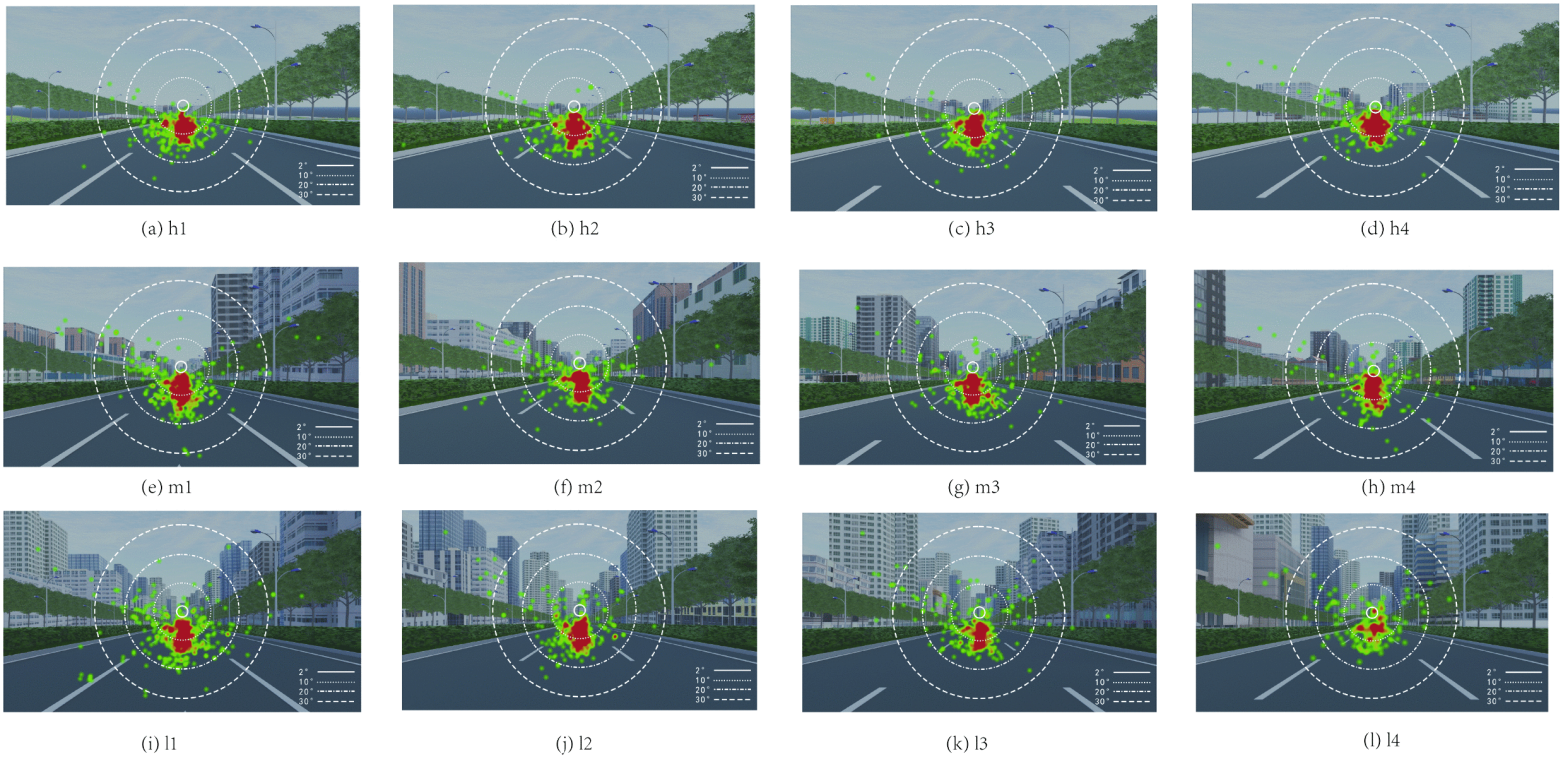
Analysis chart of average pupil change rate of drivers.

Fig 11 illustrates pronounced fluctuations in pupil diameter as drivers navigate through the M-DSVI and L-DSVI segments. Around the entry and exit points of the M-DSVI segment, frequent changes in pupil size were observed, suggesting elevated physiological arousal and tension. When combined with reaction time and fixation duration data, these fluctuations corresponded to shorter reaction times and prolonged fixations, indicating enhanced visual engagement and more efficient target recognition. These findings imply that increasing environmental complexity prompted drivers to allocate greater attentional resources in response to emerging visual challenges.

Further analysis showed that transitions into segments H2, H3, and M1 triggered sharp pupillary variations. The increased frequency of these abrupt changes correlated with heightened alertness and visual attention, indicating a greater cognitive load as an adaptive response to the complex, dynamic road conditions.

In the L-DSVI segment, both the frequency of abrupt pupil changes and the number of drivers exhibiting them increased markedly. The average amplitude of pupil dilation also rose, suggesting intensified alertness and stress responses under low DSVI conditions. These environments appear to elicit greater perceptual load, prompting stronger attentional focus and heightened psychological arousal—factors that collectively increase sensitivity to potential road hazards.

### 4.3. Driver visual load evaluation model

To quantify the impact of DSVI on drivers’ visual load and reaction capacity, this study adopted the entropy weight–weighted rank-sum ratio (EMM-WRSR) method to construct a comprehensive evaluation model. The analytical procedure was structured as follows:

(1) Indicator selection:

Five evaluation indicators were selected to capture key aspects of visual and cognitive performance, including average pupil change rate ratio, scanning speed, number of fixation points in building areas, fixation duration, average reaction time at each node. These indicators formed a data matrix comprising 12 roadway nodes and 5 evaluation indicators (Table 2).

**Table 2:** Selection of weighted rank ratio.

	Ratio of mean pupil change rate	Saccade speed (°/s)	Gaze count	Gaze duration (s)	Instant DRT (ms)
N1	0.06	280.645	5.509	1.458	522.125
N2	0.54	182.885	6.618	1.458	508.333
N3	0.59	492.309	10.364	1.291	453.166
N4	0.05	118.487	12.450	1.583	467.666
N5	0.92	280.286	14.457	1.916	516.125
N6	1.74	497.807	13.855	1.416	474.916
N7	0.35	106.581	13.882	1.583	458.500
N8	0.52	265.760	13.435	2.090	502.875
N9	0.26	210.914	15.537	2.083	536.875
N10	0.56	308.610	15.179	1.541	586.750
N11	0.51	370.032	15.925	1.695	617.791
N12	1.37	427.313	15.033	1.769	576.083

(2) Data normalization and weight calculation:

After data normalization, the weights of the five indicators were determined using the entropy weight method. The resulting distribution was as follows: pupil change rate (25.2%), scanning speed (22.0%), number of fixation points in building areas (11.3%), fixation duration (17.8%), and average reaction time (23.7%). These weights reflect the relative contribution of each indicator to the comprehensive evaluation of drivers’ visual load and reaction performance.

(3) WRSR and Probit calculation:

The WRSR and corresponding Probit values were computed to quantify the influence of road environment complexity on drivers’ visual processing capacity. The results were summarized in Table 3 and provided the basis for subsequent regression analysis and safety level classification.

**Table 3:** WRSR distribution table.

Number	WRSR distribution values	Frequency f	Cumulative frequency Σ f	R	R/n × 100%	Probit
N4	0.2372	1	1	1	8.3	3.617
N7	0.2742	1	2	2	16.7	4.033
N1	0.2993	1	3	3	25	4.326
N2	0.3075	1	4	4	33.3	4.569
N3	0.4041	1	5	5	41.7	4.79
N8	0.537	1	6	6	50	5
N9	0.5375	1	7	7	58.3	5.21
N10	0.5808	1	8	8	66.7	5.431
N11	0.5824	1	9	9	75	5.674
N5	0.5914	1	10	10	83.3	5.967
N6	0.6532	1	11	11	91.7	6.383
N12	0.7836	1	12	12	97.9	7.037

Note: Gray tables are estimated at (1–1/4 × n).

(4) Linear regression analysis:

A linear regression analysis was performed with WRSR values as the dependent variable and Probit values as the independent variable to examine the functional relationship between drivers’ visual performance scores and cumulative probability distributions. The resulting regression model was expressed as follows:


WRSR=0.389+0.169×probit (12)

The analysis showed a significance level (p = 0.000) with R² = 0.933, indicating a well-fitted model.

(5) Visual load classification:

WRSR values were categorized into three levels: low load (level 1), moderate load (level 2), and high load (level 3). This classification provided a basis for predicting drivers’ reaction capabilities and fatigue levels (Table 4).

**Table 4:** Stile-sorting critical value.

Grade	Percentile thresholds	Probit	WRSR Critical value (fitted value)
1	<15.866	<4	< 0.285
2	15.866 ~	4 ~	0.285 ~
3	84.134 ~	6 ~	0.622 ~

According to the data in Table 5, the 12 roadway nodes were ranked in descending order of their WRSR values: N12 > N6 > N5 > N11 > N10 > N9 > N8 > N3 > N2 > N1 > N7 > N4.

**Table 5:** Classification grade results.

	WRSR Rank	Probit	WRSR fitted value	Grade
N12	0.784	1	0.797	3
N6	0.653	2	0.687	3
N5	0.591	3	0.617	2
N11	0.582	4	0.567	2
N10	0.581	5	0.526	2
N9	0.538	6	0.489	2
N8	0.537	7	0.454	2
N3	0.404	8	0.418	2
N2	0.308	9	0.381	2
N1	0.299	10	0.34	2
N7	0.274	11	0.291	2
N4	0.237	12	0.221	1

In this model, higher WRSR value reflected greater visual load experienced by the driver, indicating that more attentional and cognitive effort was required to process environmental information at those location. The ranking results suggested that node N4 imposes the lowest visual load, whereas nodes N6 and N12 were associated with elevated demands, with N12 representing the highest observed level of driver visual load.

Based on the WRSR values obtained from the evaluation model, the relationship between WRSR and DSVI was further examined through linear regression analysis, which yielded the following regression equation:


WRSR=0.772−1.162×DSVI (13)

**Fig 12:**
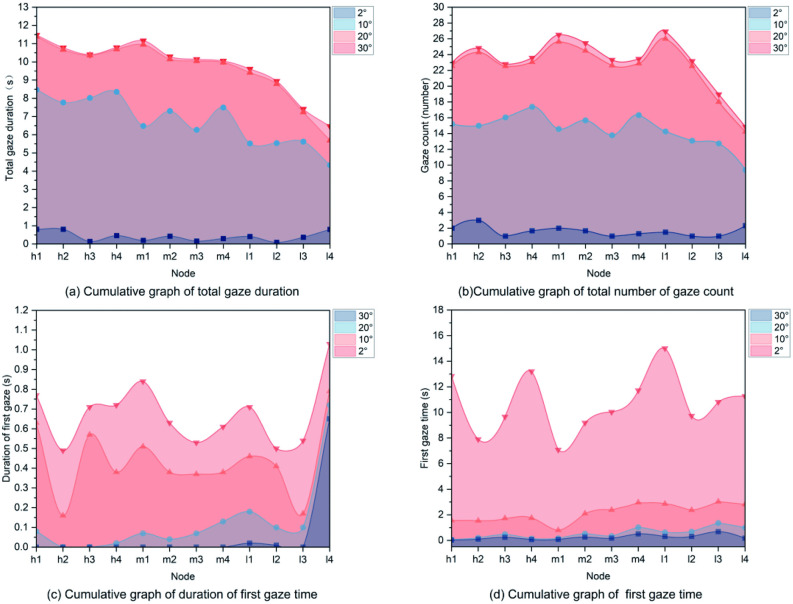
The linear regression model of WRSR and DSVI.

Through regression analysis, a valid linear model was established between DSVI (independent variable) and WRSR (dependent variable) (Fig 12). The results showed an R² of 0.641, F = 17.830, and p = 0.002 (p < 0.05), indicating a significant correlation between the two variables. Further t-test analysis of the DSVI regression coefficient yielded t = −4.223 and p = 0.002 (p < 0.01), suggesting that DSVI had a significant negative effect on WRSR.

In studies of complex real-world behaviors such as driving, a single predictor explaining over 60% of the variance is generally considered a strong effect, highlighting the value of DSVI as a key environmental metric. However, the remaining unexplained variance may be attributed to additional factors: (1) dynamic and unpredictable events such as sudden traffic conflicts or pedestrian intrusions; (2) individual differences in cognitive style and risk tolerance; and (3) long-term adaptation, whereby repeated exposure to the same route reduces perceived load.

These findings suggest that although DSVI does not provide a comprehensive explanatory model, it serves as a robust foundational metric for quantifying visual load in urban driving environments. Future research could integrate DSVI with real-time traffic data, driver physiological responses, and longitudinal behavioral observations to better capture the multifactorial determinants of driving safety and visual performance.

## 5. Conclusions and discussion

### 5.1. Main findings

This study addresses the limited research on the effects of varying roadway interface DSVI environments on driver safety and visual behavior. By analyzing drivers’ visual perception characteristics, it proposes the DSVI indicator and an evaluation model to quantify the influence of road environment complexity on driving performance. The findings contribute both to the theoretical understanding of driver-environment interaction and to provide practical applications in optimizing traffic infrastructure design and simulation-based safety evaluations.

The main conclusions are summarized as follows:

(1) Driver reaction time was significantly influenced by interface DSVI conditions. An increase in DSVI expanded the driver’s visual attention field. In M-DSVI environments, heightened driver alertness resulted in shorter reaction times, whereas L-DSVI segments were associated with increased distraction and prolonged responses, thereby compromising driving safety. These findings highlight the importance of regulating building height, setback distances, and façade treatments in urban road design to minimize visual clutter, with an optimal DSVI range identified between 0.222 and 0.397.
(2) Fixation behavior was concentrated within the 10° and 20° fields of view, which served as the primary zones for acquiring visual information. These results suggest that, in low-DSVI areas, road widening, reducing the perceptual impact of high-rise façades, and installing warning signage at critical points may help mitigate driver distraction and enhance situational awareness.
(3) A visual load model based on the entropy-weighted WRSR method confirmed a significant linear relationship between DSVI and driver visual load, with the optimal range identified as 0.241–0.397. Within this range, drivers demonstrated concentrated central gaze, stable reaction times, and moderate pupil variation, reflecting efficient visual processing. By contrast, excessively low DSVI produced information overload, whereas excessively high DSVI reduced vigilance, thereby elucidating how environmental complexity translates into changes in driving safety.

### 5.2. Policy and design implications

This study has identified an optimal DSVI range of 0.241–0.397, which offers a quantifiable benchmark for urban road planning and retrofitting. From a policy perspective, DSVI can be incorporated into urban design codes and road safety audits as a measurable indicator of spatial enclosure and openness. Specifically, the findings suggest that:

(1) Urban design regulation: To prevent excessive enclosure in low-DSVI segments (<0.241), building heights, setback distances, and façade treatments should be appropriately adjusted within planning regulations.
(2) Green infrastructure: In high-DSVI segments (>0.397), roadside trees and landscaping should be incorporated to moderate openness and sustain driver vigilance.
(3) Retrofitting existing roads: Practical interventions such as shading structures, façade treatments, or vegetation adjustments can recalibrate DSVI values toward the optimal range.
(4) Traffic safety management: DSVI thresholds can be integrated into safety audits and environmental assessments, providing engineers an evidence-based metric for evaluating and managing visual complexity.

By translating DSVI into actionable guidelines, this study bridges the gap between theoretical measurement and policy application, providing urban planners and traffic engineers with a practical tool to balance safety, comfort, and aesthetic quality in road environments.

### 5.3. Limitations and future work

Despite the valuable findings of this study, several limitations should be acknowledged. First, the analysis focused exclusively on the impact of DSVI on driver reaction time and visual characteristics and did not account for other potentially influential variables such as weather conditions, traffic flow, or driver experience. These uncontrolled variables are also likely play a significant role in shaping driver behavior and should be incorporated in future studies.

Second, the experiment was conducted in a simulated driving environment due to safety and ethical constraints. Although simulation enables controlled experimentation, it cannot fully capture the complexity and unpredictability of real-world drivings—particularly in emergencies, social interactions, or nuanced behavioral responses. As a result, the ecological validity of the findings may be limited.

Third, this study primarily examined drivers’ immediate visual and cognitive responses to changes in DSVI and did not investigate the long-term effects of sustained exposure to complex visual environments. Prolonged interactions with such environments may influence driver fatigue, adaptability, and attention strategies in ways not captured by short-term observation.

To address these limitations, future research could adopt multivariate analytical methods to comprehensively evaluate the combined effects of environmental elements such as building façades, advertisements, dynamic signage, and color schemes on driver visual perception and cognitive load. Controlled experiments could be further refined by integrating real-world driving data with simulation-based studies to enhance external validity. In addition, longitudinal studies are needed to explore how continuous exposure to low- or high-DSVI environments influences driver behavior over time. Finally, cross-cultural comparative research may provide deeper insights into how regional or cultural factors shape visual attention patterns and risk perception, thereby enhancing the applicability of DSVI-based design recommendations across diverse urban contexts.

## Supporting information

S1 File Dataset containing the raw reaction time and pupil change data. (XLSX)
